# Molecular surveillance of drug resistance: *Plasmodium falciparum* artemisinin resistance single nucleotide polymorphisms in Kelch protein propeller (K13) domain from Southern Pakistan

**DOI:** 10.1186/s12936-021-03715-0

**Published:** 2021-04-07

**Authors:** Najia Karim Ghanchi, Bushra Qurashi, Hadiqa Raees, Mohammad Asim Beg

**Affiliations:** 1 Section of Microbiology, Department of Pathology and Laboratory Medicine, Aga Khan University, Stadium Road, P.O. Box 3500, Karachi, 74800 Pakistan; 2 CRM, Aga Khan University, Karachi, Pakistan

**Keywords:** Artemisinin, *Plasmodium falciparum*, Pakistan, Drug resistance

## Abstract

**Background:**

*K13* propeller (*k13*) polymorphism are useful molecular markers for tracking the emergence and spread of artemisinin resistance in *Plasmodium falciparum.* Polymorphisms are reported from Cambodia with rapid invasion of the population and almost near fixation in south East Asia. The study describes single nucleotide polymorphisms in Kelch protein propeller domain of *P. falciparum* associated with artemisinin resistance from Southern Pakistan.

**Methods:**

Two hundred and forty-nine samples were collected from patients with microscopy confirmed *P. falciparum* malaria attending Aga Khan University Hospital during September 2015-April 2018. DNA was isolated using the whole blood protocol for the QIAmp DNA Blood Kit. The *k13* propeller gene (*k13*) was amplified using nested PCR. Double-strand sequencing of PCR products was performed using Sanger sequencing methodology. Sequences were analysed with MEGA 6 and Bio edit software to identify specific SNP combinations.

**Results:**

All isolates analysed for *k13* propeller allele were observed as wild-type in samples collected post implementation of ACT in Pakistan. C580Y, A675V, Y493H and R539T variants associated with reduced susceptibility to artemisinin-based combination therapy (ACT) were not found. Low frequency of M476I and C469Y polymorphisms was found, which is significantly associated with artemisinin resistance.

**Conclusion:**

Low frequencies of both nonsynonymous and synonymous polymorphisms were observed in *P. falciparum* isolates circulating in Southern Pakistan. The absence of known molecular markers of artemisinin resistance in this region is favourable for anti-malarial efficacy of ACT. Surveillance of anti-malarial drug resistance to detect its emergence and spread need to be strengthened in Pakistan.

**Supplementary Information:**

The online version contains supplementary material available at 10.1186/s12936-021-03715-0.

## Background

Drug resistance to anti-malarial is a major public health problem in malaria endemic countries, even with reduction in malaria cases and deaths, it is estimated that 229 million cases of malaria occurred worldwide in 2019 leading to an estimated—409,000 deaths globally [1]. Despite the significant decline in malaria morbidity and mortality over 15 years (2000–2015), a total of 413,533 confirmed malaria cases were reported in 2019 out of which 87,169 were confirmed *Plasmodium falciparum* in Pakistan [1]. According to the World Health Organization (WHO), first-line recommended treatment in Pakistan for uncomplicated *P. falciparum* malaria infections was artesunate plus sulfadoxine-pyrimethamine (AS + SP) due to high prevalence of chloroquine resistance in *P. falciparum* isolates [2].

This treatment policy has been recently revised to artemether-lumefantrine + primaquine for uncomplicated *P. falciparum* malaria [1]. Rapid emergence and spread of artemisinin resistance in *P. falciparum* is however of great concern. *Plasmodium falciparum* resistance to artemisinin is firmly established in five countries in the Greater Mekong Sub region: Cambodia, Laos, Thailand, Myanmar, and Vietnam [3, 4].

Spread of artemisinin resistance in *P. falciparum* requires effective molecular surveillance of artemisinin resistance. Recently, polymorphisms in the *k-13 propeller* gene in *P. falciparum* have been found to be a useful marker for artemisinin resistance. Single nucleotide polymorphisms (SNPs) in the *k13* region are associated with delayed parasite clearance, both in vivo and in vitro and significantly associated day 3 positive parasitaemia [3, 4]. More than 200 mutations in *k13* gene, including 108 non-synonymous variants have been reported globally [5].

Polymorphisms at nineteen loci in *k13* propeller gene (*k13*) reported from southeast Asia became a molecular signature of artemisinin resistance [6, 7]. Variants at position F446I, N485Y, Y493H, R539T, P574L and C580Y were responsible for delayed parasite clearance in patients treated with artemisinin-based combination therapy (ACT) and are, therefore, important determinants of artemisinin resistance [8, 9]. Further, genome wide association studies (GWAS) confirmed that these mutations demonstrate independent emergence at multiple geographical locations thus needs large-scale molecular surveillance [10, 11].

*k13* SNPs associated with artemisinin resistance from the China-Myanmar border, India, Bangladesh and some African countries have been reported [4, 10, 12–14]. Various non-synonymous variants associated with delayed parasite clearance have been reported from Africa in low frequencies [5]. A578S is the most common non-synonymous mutation found in parasites from five countries in sub-Saharan Africa, Western Kenya and Equatorial Guinea [15]. A578S mutation was present with a prevalence of > 1% and located close to the C580Y mutation [10].

There is a paucity of data on molecular markers of artemisinin resistance in *P. falciparum* from Pakistan. Therefore, rapid molecular surveillance of parasite populations for artemisnin resistance can help to inform the selection of drugs by control programmes. As an adjunct to in vivo drug efficacy studies, large-scale molecular surveillance of drug resistance markers is needed urgently in Pakistan. The aim of this study was to assess the genetic polymorphisms in *k13* propeller of *P. falciparum* associated with artemisinin resistance from Southern Pakistan. Molecular data collected from malaria-endemic regions will help in understanding the presence of variants in *k13* propeller gene associated with delayed parasite clearance in *P. falciparum*. The study reports *k13* propeller SNPs and *Pfmdr1* gene copy number variation in *P. falciparum* malaria cases from Southern Pakistan.

## Methods

### Study setting, participants and ethics

The study was conducted between June 2015–2018 at the Aga Khan University Hospital, a tertiary hospital located in central Karachi, and its established chain of primary health care and diagnostic service centers located in Sindh and Baluchistan provinces, Pakistan. In the study area, malaria transmission peaks during and after the monsoon season that lasts from June to October. Patients with microscopy confirmed *P. falciparum* mono-infection were eligible for enrolment irrespective of age, gender and disease severity.

The study was conducted in accordance with the Declaration of Helsinki and Good Clinical Practice [16]. Informed consent was obtained from all participants or in case of children from their parents/legal guardians. The study was approved by the ethical review committee of Aga Khan University Hospital, Karachi, Pakistan.

### Blood collection and microscopy

Two ml of intravenous blood were collected in an EDTA tube from all patients suspected of malaria referred to the laboratory for investigation of malaria infection. For screening purposes, a thick blood film was prepared and analysed using Leishman stain according to routine laboratory practice. In case of a positive screening result, a thick and thin Giemsa-stained blood film was prepared for confirmation of the presence of malaria parasites and species identification. For all patients with confirmed *P. falciparum* mono-infection the parasite density was assessed by counting asexual parasites against 200 white blood cells (WBC) on the thick film and quantified (parasites/µl) by assuming an average of 8000 WBC per μl blood [17]. All blood slides were examined by experienced microscopist at the clinical laboratory of Aga Khan University Hospital. For quality control, 10% of the blood slides were re-examined by an independent microscopist unaware of the initial result.

The remaining blood was transferred to cryovials and kept frozen at −80ºC until used for DNA extraction. A brief epidemiological and demographic history was also collected from each participant using a structured questionnaire.

### DNA extraction and PCR genotyping of *k13* propeller domain

DNA was extracted using Qiamp DNA mini Kits (Qiagen, USA) from 200 µl of whole blood as per manufacturer’s instructions. Extracted DNA was stored at – 20 °C until amplified by PCR.

The *k13* propeller domain was amplified by nested PCR described elsewhere [4]. Briefly, using the following primers: for the primary PCR (kelch-out-f 5′CGGAGTG ACCAAATCTGGGA-3′ and kelch-out-r 5′GGGAATCTGGTGGTAACAGC-3′) and the nested PCR (kelch-in1-r 5′GCCTTGTTGAAAGAAGCAGA-3′, kelch-in1-f 5′-GCCAAGCTGCCATTCATTTG-3′ kelch-in-f_5′CGCCAGCATTGTTGACTAAT-3′ and kelch-in-r 5′GCGGAAGTAGTAGCGAGAAT-3′). The size of nested PCR products were 1312 and 849 bp corresponding to nucleotide 101–1412 and 1279–2127 (representing codons 427–709) of PF3D7_1343700. *k13* propeller domain representing codons 427–709 included variants related to delayed parasite clearance. PCR products were resolved on 2% agarose gels (Amresco, Solon, OH) visualized under UV transillumination (GelDoc^®^, Biorad, Hercules, CA, USA) (Additional file 1).

Secondary PCR products were purified by ExoSAP-IT (Affymetrix, Santa Clara, CA, USA) and sequenced commercially (Macrogen Inc. Seoul, Korea). The primers for sequencing were same with those of nested PCR.

*Pfmdr1* gene copy number variation polymorphism was determined using real time PCR (ABI Prism^®^ 7500) as previously described [18]. All samples were run in duplicates. The clones, 3D7 and K1 were used as single copy calibrators and FCB and Dd2 represented multiple copy controls. *Pfmdr1* copy numbers were calculated using a comparative threshold method (ΔΔCt method).

### Statistical analyses

Data were entered, validated and analysed using SPSS version 16.0. Molecular Evolutionary Genetics Analysis (MEGA) software version 10 was used to analyse the sequences using the 3D7 clone sequence (PF3D7_1343700 K13) obtained from NCBI database as a reference [19] (Additional file 2). SNP proportions were calculated as the number carrying a certain resistant allele divided by the number of samples with positive PCR outcome.

## Results

A total of 1016 patients with malaria were included in this study and two hundred and forty-nine were microscopy and PCR confirmed for *P. falciparum* mono-infection, remaining were confirmed as *P. vivax* malaria. Patients from all age groups were infected and there was no significant difference among pediatrics and adult population. The demographic of the patients are presented in Table 1. A total of 241 (97%) sequence from *k13* propeller PCR products were obtained. *K13* propeller gene sequences from study isolates were compared with the reference 3D7 strain (PF3D7_1343700) retrieved from Gene bank.

**Table 1:** Demographic characteristics of enrolled patients

	All [n = 1016]	*P.vivax* [n = 767]	*P. falciparum* [n = 249]
Age^a^			
≤ 5 years	68 [6.69%]	46 [5.99%]	22 [8.8%]
6–15 years	137 [13.4%]	97 [12.6%]	40 [16.0%]
> 15 years	811 [79.8%]	624 [81.3%]	187 [75.1%]
Sex^a^			
Male	705 [69.3%]	529 [68.9%]	176 [70.6%]
Female	311 [30.6%]	238 [31.0%]	73 [29.3%]
Parasite density^b^ [parasites/µl]	11,100 [80–540000]	12,720 [80–540000]	7480 [80–126000]

^a^Number of patients are presented with proportions in brackets

^b^Parasite densities were available form 216 patients. Median data are presented with range in brackets

Substitutions at positions Y493H, F446I, R539T, R561H, P574L and C580Y were not detected in this study isolates. M476I was observed in was observed in 3 isolates none of the patients had history of travel and recovered from malaria post treatment. C469Y was observed in one isolate. Nonsynonymous novel mutations at positions T508N and S577L were observed in 12 (5%) and 13 (5%) isolates, respectively. These novel mutations were associated with delayed parasite clearance or treatment failures in the study subject. Low frequency of K189T mutation outside *k13* propeller region was reported in this study (Table 2). No *k13* propeller validated variants were detected at positions F446I, Y493H, G538V, R539T, P553L, R561H, V568G, P574L, C580Y and A675V in this study. Double mutations were not observed in any isolate, all the samples tested showed single *k13* variant. The most common *k13* haplotype based on globally reported 19 SNPs was wild type in this study. The wild type haplotype defined as FGNSCAFLYGPEPACVEAA at amino acid positions 446, 450, 458, 459, 469, 481, 483, 492, 519, 533, 556, 574, 578, 580, 581, 668, 675 and 676. Amplification in *pfmdr1* gene copy number was not observed in the study isolates.

**Table 2:** Frequency of *k13* propeller variants observed in this study

Variant types	Single nucleotide polymorphism	Base substitution	Variant Frequency	Wild type	Status	Variant type
Wild type	–	–	–	241 (1)	–	WT
K189T	c.566A > C	A**A**A > A**C**A	1 [0.004]	240 [0.99]	–	NS
C469Y	c.1407C > G	TG**C** > TG**G**	1 [0.004]	240 [0.99]	Candidate	NS
M476I	c.1428G > T	AT**G** > AT**T**	3 [0.01]	238 [ 0.98]	Validated	NS
F483I	c.1447T > A	**T**TT > **A**TT	9 [0.04]	232 [0.96]	–	NS
S485N	c.1454G > A	A**G**T > A**A**T	7 [0.03]	234 [0.97]	–	NS
F491F	c.1473F > F	TT**C** > TT**T**	52 [0.22]	189 [0.78]	–	S
L492L	c.1476A > G	TT**A** > TT**G**	2 [0.01]	239 [0.99]	–	S
F506V	c.1516 T > G	**T**TT > **G**TT	8 [0.03]	232 [0.96]	–	NS
T508N	c.1523C > A	A**C**T > A**A**T	12 [0.05]	229 [0.95]	–	NS
S577L	c.1730S > L	T**C**A > T**T**A	13 [0.05]	228 [0.95]	–	NS

*NS* nonsynonymous, *S* synonymous, [–] variants of unknown significance or not reported

*k13* variants that are either validated or are associated/candidates for ART resistance are shown

## Discussion

This study reports survey of *k13* propeller polymorphisms in *P. falciparum* from southern Pakistan majority of samples were from metropolitan of Karachi with a population of more than 16 million. Candidate variants in the *k13* gene N458Y, F466I, Y493H, R539T, P574L and C580Y, which were associated with delayed parasite clearance and prolonged ex vivo parasite survival in Southeast Asia were not observed [4, 8].

K189T is a common mutation observed in upstream region of *k13* gene. It has not been associated with artemisnin resistant clinical phenotype [14, 20]. Low proportion of K189T was found in this study. Low frequencies of K189T has also been reported from Bangladesh and China-Myanmar border in comparison to a higher prevalence in Africa. In addition, K189T mutation was reported from India with a comparatively higher frequency, however no correlation with artemisnin resistance was observed [21].

Low frequency of M476I with no clinical evidence of artemisnin resistance was observed in this study. M476I variant has been associated with artemisinin tolerance in vitro in the Tanzanian parasite population and is a validated marker of artemisinin resistance. This finding is alarming and suggest that larger scale monitoring may be urgently required. These variants in *k13* gene have also previously been reported from southern Myanmar, South Vietnam, Bangladesh and India [14, 22]. C469Y is known to be associated with artemisinin tolerance from China-Myanmar border and Uganda is reported in one isolate in this study [3, 23].

Variants associated with artemisnin resistance in South East Asia were not detected in Africa suggesting independent origin of mutation in different regions [10, 24, 25]. V566I and A578S are most notable of seven unique nonsynonymous variants reported from sub-Saharan Africa which were not observed in Southeast Asia [26]. A578S has been commonly reported from Africa, India and Bangladesh with no clinical evidence of artemisinin resistance [5, 21].

F466I, Y493H, R539T, P574L and C580Y variants have reached intermediate to fixation status in South East Asia and China where currently artemisinin resistance is confined. These variants confer resistance in an artemisinin-resistant parasite line selected in the laboratory and are associated with delayed parasite clearance in clinical isolates [4, 27]. F446I which is highly prevalent in China-Myanmar and Northern Myanmar and has been associated with prolonged parasite clearance recently was not reported in this study [28, 29]. C580Y is highly prevalent in Cambodia, Thailand and Myanmar. The frequency of the C580Y allele increased significantly in two western provinces of Cambodia and it became near fixation in these areas. Association of N458Y with artemisinin resistance was inconsistently reported from the Thai- Myanmar border region. Y493H and C580Yvariants reportedly originating from Cambodia have subsequently spread throughout Vietnam [6].

Single nucleotide polymorphism in *k13* conferring drug resistance have emerged independently and differ regionally [4, 10]. In recent study it has been noted that validated and candidate *k13* mutation may not confer artemisinin resistance in isolation but would act in presence of multiple variant which were noted in African and Southeast Asian parasite populations [27, 30].

In this study, low frequency of *k13* propeller mutations were observed and no previously validated artemisinin resistance variant reported from South East Asia were found. A majority of isolates in this study carried wild-type *k13* propeller gene corresponds to haplotype FGNSCAFLYGPEPA-CVEAA [31]. Synonymous and nonsynonymous substitutions of unclear phenotype were identified. Recent studies from Pakistan also reported a lack of *k13* variants associated with artemisinin resistance. Both studies, however reported synonymous and non- synonymous with no or limited association with artemisnin resistance consistent with current findings [32, 33]. These results are encouraging and suggest that artemisinin resistance is not yet established in Southern Pakistan. However, presence of low frequency of M476I and evolving variants is however alarming. This study shows there may be regional variation in mutation profile and therefore it is important that consistent and regular molecular surveillance monitoring is conducted throughout Pakistan.

Although critical SNPs associated with artemisinin resistance in SE Asia were not detected in this study and concur with African studies which report different mutation [24, 25]. Novel *k13* propeller coding substitutions T508N and S57L were reported in this study. The phenotypes of these coding polymorphisms remain unclear and will require further characterization to better characterize the clinical impact on artemisinin resistance in Southern Pakistan.

This study confirms that recently revised treatment option from AT + SP to AL for uncomplicated *P. falciparum* malaria should be an effective regimen as *pfmdr1* gene amplifications were not observed. *Pfmdr1* gene copy number amplification has been associated with an increased risk for treatment failure after mefloquine monotherapy and artesunate-mefloquine combination therapy [18]. Presence of single copy *pfmdr 1* gene is consistent with previously reported studies from Pakistan [2]. The low prevalence of *pfmdr1* amplifications observed in this study suggests that both artesunate-mefloquine and artemether-lumefantrine combination would be efficacious in Southern Pakistan.

One of the limitation of the present study was absence of availability of coordinated clinical follow up, patient outcomes and molecular data to provide better understanding of the biological and clinical impact of these unique genotypes. Complementing these studies with ongoing, large-scale molecular epidemiologic surveillance will enhance ability to monitor artemisinin resistance in Pakistan. Integration of these efforts in the national malaria control programme may help forestall the spread of resistance and enhance the global durability of artemisinin therapies.

The absence of known molecular markers of artemisinin resistance in this region is favourable for the anti-malarial efficacy of ACT. However, presence of M476I in low frequency is matter of concern in Pakistan. It is difficult to predict how soon resistance variants may appear in absence of wide scale molecular surveillance. Validated data on *k13* mutation are known to have population based specificity therefore data from Pakistan has critical implications for implementation of appropriate treatment policy and development of elimination strategy. Molecular surveillance can provide a framework to rapidly monitor for the emergence or importation of resistance alleles.

## Supplementary Information

**Additional file 1:** Primers.

**Additional file 2:** Genebank submission record.

## Data Availability

The datasets used and analysed during the current study are available from the corresponding author on reasonable request. Most of data analysed during this study are presented in this published article.
