# Supplementary material for: Molecular surveillance of drug resistance: Plasmodium falciparum artemisinin resistance single nucleotide polymorphisms in Kelch protein propeller (K13) domain from Southern Pakistan
Source: Malar J. 2021 Apr 7;20:176. doi: 10.1186/s12936-021-03715-0 (PMC8028081; doi:10.1186/s12936-021-03715-0)

**>XM_001350122.1 Plasmodium falciparum 3D7 kelch protein K13 (PF3D7_1343700), partial mRNA**

ATGGAAGGAGAAAAAGTAAAAACAAAAGCAAATAGTATCTCGAATTTTTCTATGACGTATGATAGGGAAT

CTGGTGGTAACAGCAATAGTGATGATAAAAGCGGAAGTAGTAGCGAGAATGATTCTAATTCATTTATGAA

TCTAACTAGTGATAAAAATGAGAAAACGGAAAATAATAGTTTCCTTTTAAATAATAGTAGTTATGGAAAT

GTTAAAGATAGCCTATTAGAATCCATTGATATGAGTGTATTAGATTCGAACTTTGATAGTAAAAAAGATT

TTTTACCAAGTAATTTATCAAGAACATTTAATAATATGTCTAAAGATAATATAGGAAATAAATATTTAAA

TAAATTGTTAAATAAAAAAAAAGATACTATTACAAATGAAAATAATAATATTAATCATAATAATAATAAT

AATAATCTGACAGCAAATAATATAACTAATAATCTTATTAATAATAATATGAATTCTCCATCAATTATGA

ATACCAACAAAAAAGAGAATTTTTTAGATGCAGCAAATCTTATAAATGATGATTCTGGATTAAACAATTT

AAAAAAATTTTCAACTGTAAATAATGTAAATGATACTTATGAAAAGAAAATTATTGAAACGGAATTAAGT

GATGCTAGTGATTTTGAAAATATGGTAGGTGATTTAAGAATTACATTTATTAATTGGTTAAAAAAGACAC

AAATGAATTTTATTCGAGAAAAAGATAAATTATTTAAAGATAAGAAAGAACTAGAAATGGAAAGAGTACG

ATTGTACAAAGAATTAGAAAACCGTAAAAATATTGAAGAACAGAAATTACATGATGAAAGAAAGAAATTA

GATATTGATATATCTAATGGTTATAAACAAATAAAAAAAGAAAAAGAAGAACATAGGAAACGATTTGATG

AAGAAAGATTAAGATTTTTACAAGAAATCGATAAAATTAAATTAGTATTATATTTAGAAAAAGAAAAATA

TTATCAAGAATATAAAAATTTTGAGAATGATAAAAAAAAAATTGTTGATGCAAATATTGCTACTGAAACT

ATGATTGATATTAATGTTGGTGGAGCTATTTTTGAAACATCTAGACATACCTTAACACAACAAAAAGATT

CATTTATAGAGAAATTATTAAGTGGAAGACATCATGTAACCAGAGATAAACAAGGAAGAATATTCTTAGA

TAGGGATAGTGAGTTATTTAGAATTATACTTAACTTCTTAAGAAATCCGTTAACTATACCCATACCAAAA

GATTTAAGTGAAAGTGAAGCCTTGTTGAAAGAAGCAGAATTTTATGGTATTAAATTTTTACCATTCCCAT

TAGTATTTTGTATAGGTGGATTTGATGGTGTAGAATATTTAAATTCGATGGAATTATTAGATATTAGTCA

ACAATGCTGGCGTATGTGTACACCTATGTCTACCAAAAAAGCTTATTTTGGAAGTGCTGTATTGAATAAT

TTCTTATACGTTTTTGGTGGTAATAACTATGATTATAAGGCTTTATTTGAAACTGAGGTGTATGATCGTT

TAAGAGATGTATGGTATGTTTCAAGTAATTTAAATATACCTAGAAGAAATAATTGTGGTGTTACGTCAAA

TGGTAGAATTTATTGTATTGGGGGATATGATGGCTCTTCTATTATACCGAATGTAGAAGCATATGATCAT

CGTATGAAAGCATGGGTAGAGGTGGCACCTTTGAATACCCCTAGATCATCAGCTATGTGTGTTGCTTTTG

ATAATAAAATTTATGTCATTGGTGGAACTAATGGTGAGAGATTAAATTCTATTGAAGTATATGAAGAAAA

AATGAATAAATGGGAACAATTTCCATATGCCTTATTAGAAGCTAGAAGTTCAGGAGCAGCTTTTAATTAC

CTTAATCAAATATATGTTGTTGGAGGTATTGATAATGAACATAACATATTAGATTCCGTTGAACAATATC

AACCATTTAATAAAAGATGGCAATTTCTAAATGGTGTACCAGAGAAAAAAATGAATTTTGGAGCTGCCAC

ATTGTCAGATTCTTATATAATTACAGGAGGAGAAAATGGCGAAGTTCTAAATTCATGTCATTTCTTTTCA

CCAGATACAAATGAATGGCAGCTTGGCCCATCTTTATTAGTTCCCAGATTTGGTCACTCCGTTTTAATAG

CAAATATATAA

**PCR PRIMERS:**

**For Diagnostic PCR**

| Common Reverse | (5'-GTATCTGATCGTCTTCACTCCC) |
| --- | --- |
|  |  |
| P.falciparum | (5'-AACAGACGGGTAGTCATGATTGAG) |
|  |  |
| P. vivax | (5'-CGG CTTGGAAGTCCTTGT) |

**FOR KP13:**

| K13_PCR_F | CGGAGTGACCAAATCTGGGA |
| --- | --- |
| K13_PCR_R | GGGAATCTGGTGGTAACAGC |
| K13_N1_F | GCCAAGCTGCCATTCATTTG |
| K13_N1_R | GCCTTGTTGAAAGAAGCAGA |
| K13_N2_F | CGCCAGCATTGTTGACTAAT |
| K13_N2_R | GCGGAAGTAGTAGCGAGAAT |

1. Ariey F, Witkowski B, Amaratunga C, Beghain J, Langlois AC, Khim N, Kim S, Duru V, Bouchier C, Ma L, et al: **A molecular marker of artemisinin-resistant Plasmodium falciparum malaria.** *Nature* 2014, **505:**50-55.


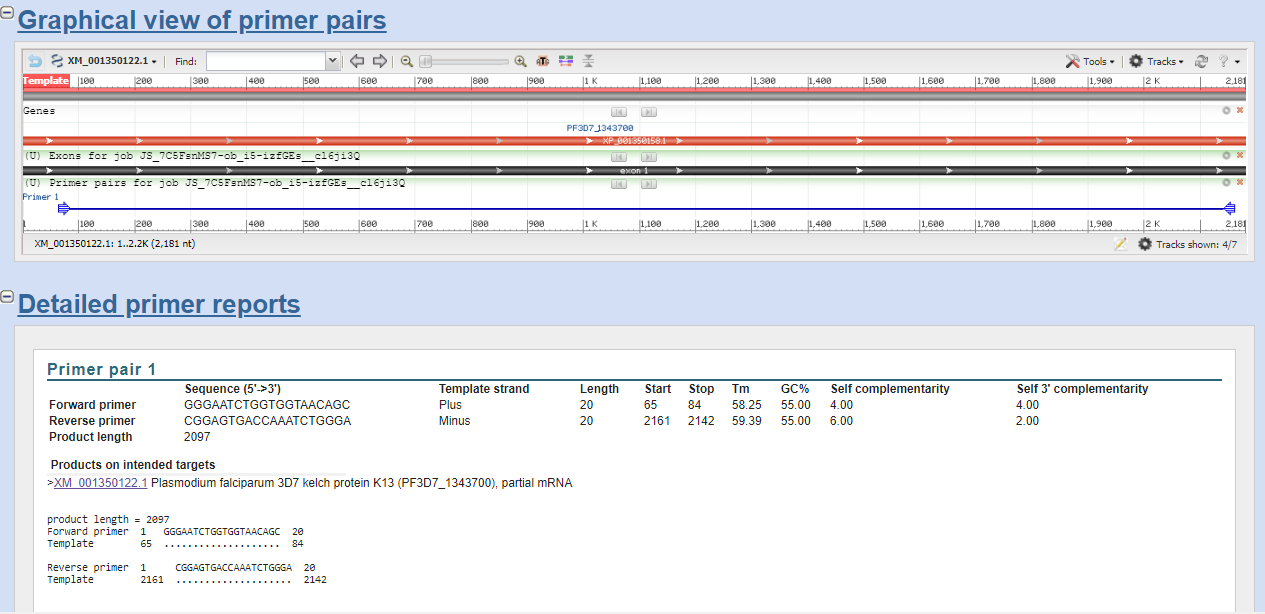


| P.falciparum | (5'-AACAGACGGGTAGTCATGATTGAG) |
| --- | --- |

Blast Report:


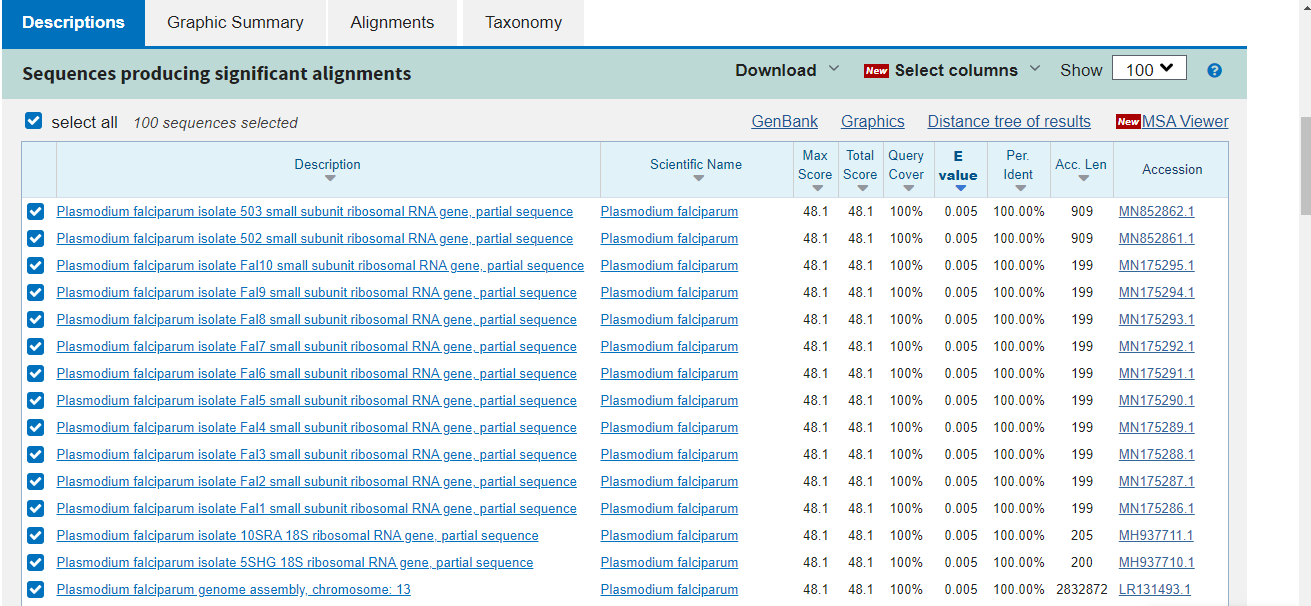


| P. vivax | (5'-CGG CTTGGAAGTCCTTGT) |
| --- | --- |


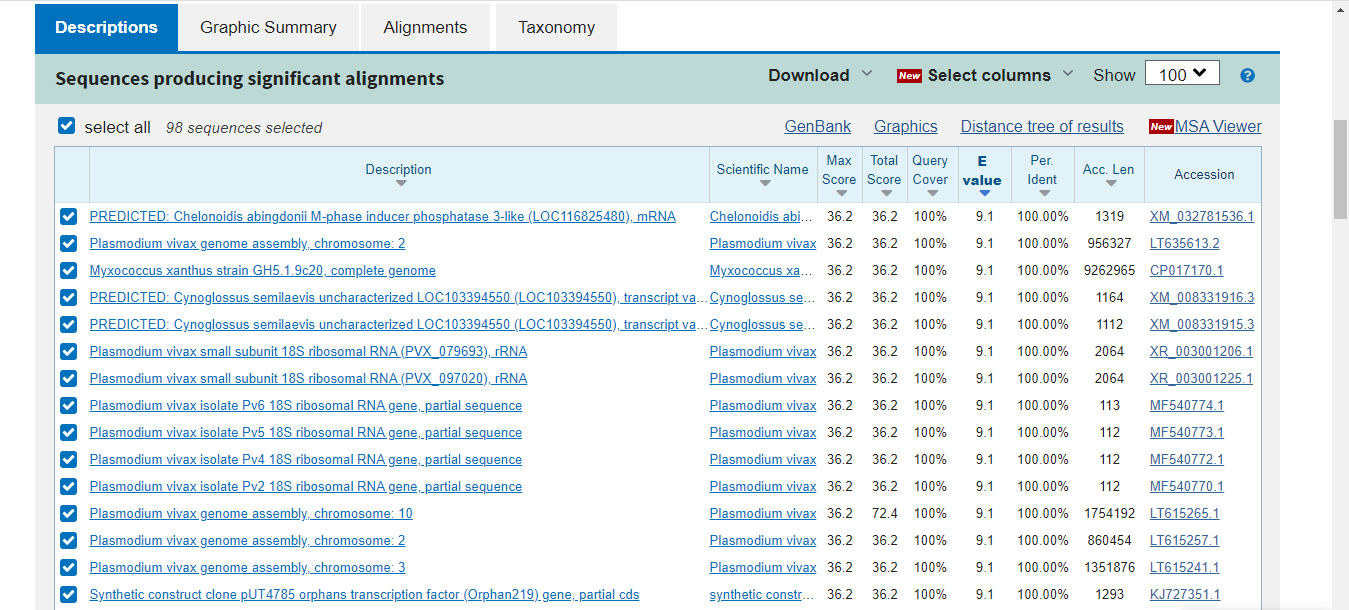


| Common Reverse | (5'-GTATCTGATCGTCTTCACTCCC) |
| --- | --- |


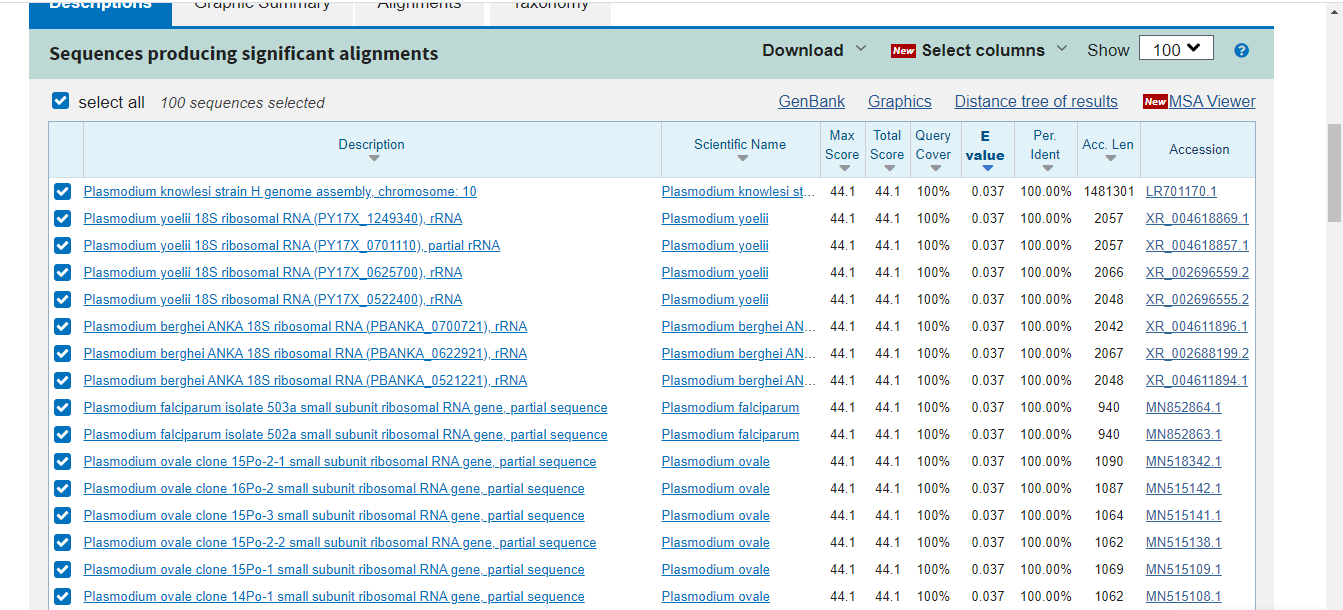

Supplement: Supplementary file 1 — Additional file 1: Primers. [file 12936_2021_3715_MOESM1_ESM.docx]
